# Supplementary material for: Higher carbon sequestration potential and stability for deep soil compared to surface soil regardless of nitrogen addition in a subtropical forest
Source: PeerJ. 2020 May 11;8:e9128. doi: 10.7717/peerj.9128 (PMC7224229; doi:10.7717/peerj.9128)
Supplement: Supplemental Information 1 — Figures include the soil fractionation scheme, the cumulative released C, and the DOC and its isotope signature. [file peerj-08-9128-s001.docx]

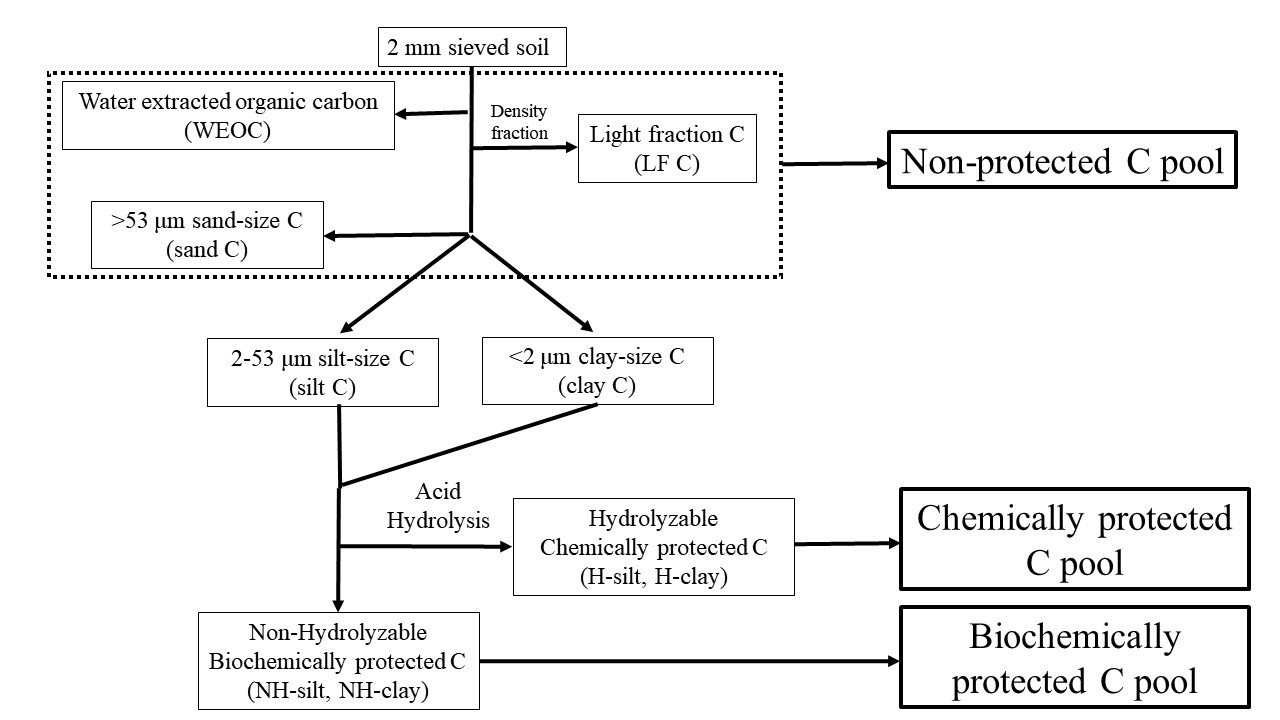


**Fig. S1** A soil fractionation scheme that isolates SOC into seven fractions and grouped into three functional C pools: non-protected C pool (WEOC, LF and sand C), chemically protected C pool (H-silt + H-clay), and biochemically protected fractions C pool (NH-silt + NH-clay). WEOC: water extracted organic carbon, LF: light fraction C, NH-silt: non-hydrolyzable silt C, NH-clay: non-hydrolyzable clay C, H-silt: hydrolyzable silt C, H-clay: hydrolyzable clay C





**Fig. S2.** The effects of glucose and N addition on cumulative released C, cumulative released C derived from glucose and cumulative primed C during the 20-day incubation in surface soil (A, C, E) and deep soil (B, D, F)





**Fig. S3** The effects of glucose and N addition on δ^13^C of DOC (A) and glucose-derived DOC (B) after 20 days incubation. The lowercase letter indicated the difference among the different treatments in the same soil depth. These values are means ± SE (n = 3). DOC: the dissolved organic carbon extracted by 0.05 mol L^-1^ K_2_SO_4_ from fresh soil samples at the end of the 20 days incubation.

**Table S1** The effect of soil depth and N addition on the relative PE, the C sequestration potential and net C sequestration potential

| Parameters | Nitrogen | Depth | Nitrogen*Depth |
| --- | --- | --- | --- |
| Relative PE (%) | *** | *** | ns |
| The C sequestration potential (%) | ns | *** | ns |
| Net C sequestration potential (%) | *** | *** | * |

* *p*<0.05; *** *p*<0.001; ns: not significant.

**Table S2** The distribution of new C in the measured soil fractions after 20 days incubation

| SOC fractions | Treatments | New C% in bulk new C | |
| --- | --- | --- | --- |
|  |  | Surface soil | Deep soil |
| WEOC | Glu | 8.2±0.1Aa* | 4.9±0.1Ba* |
|  | Glu+N | 8.7±0.1Ab* | 5.3±0.3Ba* |
| LF | Glu | 6.1±0.3Aa* | 1.8±0.1Ba* |
|  | Glu+N | 7.7±0.9Aa | 2.0±0.3Ba* |
| Sand | Glu | 0.5±0.1Aa* | 0.9±0.3Aa |
|  | Glu+N | 0.9±0.1Ab* | 1.9±0.1Bb* |
| H-silt | Glu | 43.0±0.5Aa* | 41.6±1.0Aa* |
|  | Glu+N | 41.0±0.6Aa* | 38.7±2.1Aa* |
| NH-silt | Glu | 10.2±0.2Aa* | 3.5±0.3Ba* |
|  | Glu+N | 12.0±0.8Aa* | 4.0±0.2Ba* |
| H-clay | Glu | 17.2±0.2Aa* | 34.2±1.8Ba* |
|  | Glu+N | 16.3±0.3Aa* | 34.9±2.1Ba* |
| NH-clay | Glu | 4.4±0.3Aa* | 2.7±0.2Ba* |
|  | Glu+N | 5.5±0.3Ab* | 3.8±0.2Bb* |

For the same C fraction, different lowercase indicates a significant difference between Glu (single glucose addition) and Glu+N (glucose plus N addition) treatment within the same soil depth, and different capital letters indicate a significant difference between surface soil and deep soil within the same treatment. * indicated that the incorporation proportion of new C in the fraction was significantly different with the native C. These values are means ± SE (n = 3).
